# Pyrosequencing of *Mytilus galloprovincialis* cDNAs: Tissue-Specific Expression Patterns

**DOI:** 10.1371/journal.pone.0008875

**Published:** 2010-01-25

**Authors:** John A. Craft, Jack A. Gilbert, Ben Temperton, Kate E. Dempsey, Kevin Ashelford, Bela Tiwari, Tom H. Hutchinson, J. Kevin Chipman

**Affiliations:** 1 Biological and Biomedical Sciences, Glasgow Caledonian University, Glasgow, United Kingdom; 2 Plymouth Marine Laboratory, The University of Liverpool, Liverpool, United Kingdom; 3 School of Biological Sciences, The University of Liverpool, Liverpool, United Kingdom; 4 Natural Environment Research Council Environmental Bioinformatics Centre, Centre for Ecology and Hydrology, Wallingford, United Kingdom; 5 School of Biological Sciences, University of Birmingham, Birmingham, United Kingdom; Cairo University, Egypt

## Abstract

**Background:**

Mytilus species are important in marine ecology and in environmental quality assessment, yet their molecular biology is poorly understood. Molecular aspects of their reproduction, hybridisation between species, mitochondrial inheritance, skewed sex ratios of offspring and adaptation to climatic and pollution factors are priority areas.

**Methodology/Principal Findings:**

To start to address this situation, expressed genetic transcripts from *M. galloprovincialis* were pyrosequenced. Transcripts were isolated from the digestive gland, foot, gill and mantle of both male and female mussels. In total, 175,547 sequences were obtained and for foot and mantle, 90% of the sequences could be assembled into contiguous fragments but this reduced to 75% for the digestive gland and gill. Transcripts relating to protein metabolism and respiration dominated including ribosomal proteins, cytochrome oxidases and NADH dehydrogenase subunits. Tissue specific variation was identified in transcripts associated with mitochondrial energy metabolism, with the digestive gland and gill having the greatest transcript abundance. Using fragment recruitment it was also possible to identify sites of potential small RNAs involved in mitochondrial transcriptional regulation. Sex ratios based on Vitelline Envelop Receptor for Lysin and Vitelline Coat Lysin transcript abundances, indicated that an equal sex distribution was maintained. Taxonomic profiling of the *M. galloprovincialis* tissues highlighted an abundant microbial flora associated with the digestive gland. Profiling of the tissues for genes involved in intermediary metabolism demonstrated that the gill and digestive gland were more similar to each other than to the other two tissues, and specifically the foot transcriptome was most dissimilar.

**Conclusions:**

Pyrosequencing has provided extensive genomic information for *M. galloprovincialis* and generated novel observations on expression of different tissues, mitochondria and associated microorganisms. It will also facilitate the much needed production of an oligonucleotide microarray for the organism.

## Introduction

The common blue mussels belong to the Mytilus genus (*M. edulis, M. galloprovincialis, M. trossulus*), are found world-wide and play a significant role in coastal ecology. Although the Mytilidea are a subject of much continuing research, there are many aspects of their biology which require elucidation. For instance they show species-selective distribution patterns: In general, *M. edulis* is found in Northern latitudes (e.g. Scotland, Northern/Mid England) while *M. galloprovincialis* is found further south (e.g. parts of predominantly Southern England, Atlantic France and the Mediterranean) [1]–[3]. However, the distribution is mosaic, with hybridisation occurring between species at the boundaries of each range [1]. Currently, it is not clear what controlling factors maintain the separate populations. Another curious feature of Mytilus biology concerns the manner of inheritance of the mitochondrial genome. Females inherit in the expected matrilineal way but some males inherit from both parents, a phenomenon termed Doubly Uniparental Inheritance [4], [5]. The mechanism of DUI appears to be associated with reproductive biology in which gender balance of offspring can be highly skewed to male or female by an unknown maternal determinant [6], [7].

For many years mussel species have been used to monitor the quality of the aquatic environment in relation to the impact of pollutants (e.g. “Mussel Watch”) [8]. The organisms are particularly useful in this context because they inhabit regions of differential pollution status, they accumulate xenobiotics and they are sessile. To date the end-points used for monitoring effects in mussels are based on a small number of specific biomarkers such as genotoxicity end points [9] or “scope for growth measurements” [10].

In view of the ecological importance of *Mytilus* to the marine environment, understanding susceptibility to pollutants, hybridisation and differential stress resistance is important especially during an era of climate change and pollution incidents. Understanding their biology depends on defining basic processes such as toxic responses, reproduction, speciation mechanisms and adaptation to stressors. These processes will be more readily addressed if the transcriptomes of Mytilus species are available. Proof of principle of the use of transcriptomics for non-model organisms has been demonstrated in determination of responses of e.g. aquatic organisms to toxicants [11], [12] and the identification of geographic sites of origin [13]. Whilst there have been some studies on transcriptomic responses of the mussel to toxicant exposure [14], [15], the genomic resources for those studies have been extremely limited. There is an ongoing genome project studying *M. californianus* that has deposited 45000 EST sequences at GenBank but this species is less relevant to Atlantic, Baltic and other Northern hemisphere environments. Other initiatives have accumulated a few thousand EST sequences for *M. edulis* [16], [17] and rather more for *M. galloprovincialis* [18], [19]. MytiBase (http://mussel.cribi.unipd.it/) contains clusters of *Mytilus galloprovincialis* ESTs with consensus sequences annotated by Blast and InterProScan.

All existing published approaches to discovery of the Mytilus transcriptome are based on classical cloning and Sanger sequencing strategies. The work described here is the first use of pyrosequencing [20] with a mollusk. This approach allows faster sequencing output and increased coverage. In this initial study the EST sequences were determined in four tissues for *M. galloprovincialis*. Comparative analyses of tissue-specific transcripts have informed on several aspects of Mytilus biology and the extensive datasets will open new lines of investigation of mussel biology.

## Results

### Animal Characterization and Summary Statistics for Pyrosequencing

**Figure 1:**
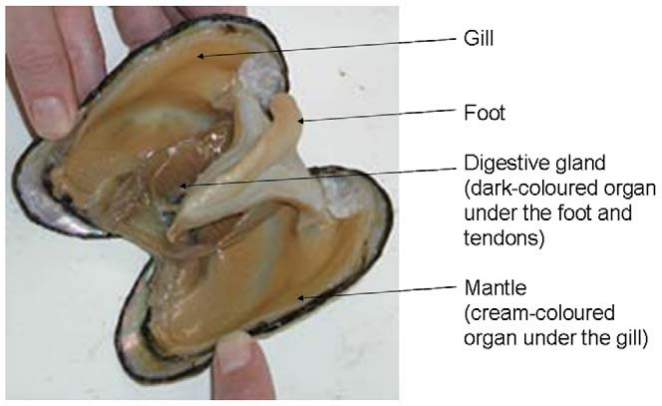
Anatomy of the mussel.

Mussels were collected from Port Quin, Cornwall, UK and four tissues (mantle, digestive gland, gill and foot (Fig. 1)) dissected from each animal. The species-identity of the animals collected from Port Quin was established using the Me-15 and -16 primers for the Glu gene. Previous investigations [21] have shown 100% identity of mussels as *M. galloprovincialis* at this sampling site with no evidence of hybrids with *M. edulis*. PCR products were analysed on a 2% agarose gel yielding species-specific bands at 180 base-pairs (bp) for *M. edulis*, obtained from the Firth of Clyde and run as a control, and 126 bp for the animals from Port Quin and thus *M. galloprovincialis*. The gender of animals was ascertained by determination of the sex-specific expression of VERL and VCL transcripts in mantle and the ratio of male to female animals was similar.

Total RNA was isolated and pooled from an equal number of male and female animals and used for the synthesis of cDNA libraries from each tissue. These were then pyrosequenced and Table 1 shows summary statistics of the reads for each sample. In total, 175,547 pyrosequencing reads were obtained constituting a total of 34,424,691 bp. Average read length across all tissue samples was 196 bp. Following the separate assembly of the sequences from each sample, a total of 8,586 contigs were obtained of which 223 had lengths longer than 500 bp and 1023 could be annotated to known function. The average number of singletons (fragments which could not be assembled into any contiguous sequence) comprised ∼10% of each dataset, with the exception of the digestive gland and gill datasets, in which singletons comprised as much as 25%. This level of assembly suggests relatively high transcriptome coverage, although without information on the average number of transcripts per cell in each tissue, this cannot be confirmed. The digestive gland and gill from *M. galloprovincialis* showed potentially lower coverage (i.e. a greater proportion of singletons) but also produced the greatest number of large contigs, with 174 of the 6,816 assembled contigs for these tissues having lengths >500 bp - four times greater than the total number of contigs from the foot and mantle. This may be indicative of a small proportion of highly abundant transcripts from an otherwise highly diverse transcript pool, which may be higher in the digestive gland and gill due to their more active role in homeostatic mechanisms responding to food and the environment, when compared with the foot or mantle.

**Table 1:** Summary statistics for pyrosequencing of ESTs from various tissues of Mytilus galloprovincialis.

Species	Mytilus galloprovincialis			
Tissue	D. gland	Foot	Gill	Mantle
Total No. of reads	33,992	31,227	58,271	52,057
Total size (bp)	6,988,845	5,984,748	12,157,845	9,293,253
Average length (bp)	205	191	208	178
Total No. of contigs	2,103	724	4,713	1,046
Total No. of contigs with functional assignment against nr	407	145	320	151
Contigs >500 bp	78	24	96	25
Total reads within contigs	25,428	28,682	44,178	46,180
Singleton reads	8,564 (25%)	2,545 (8%)	14,093 (24.2%)	5,877 (11.3%)

D.gland–digestive gland. Bp–base pairs. Contigs–assembled contiguous fragments. Nr–NCBI non-redundant protein database.

### Tissue- and Species-Specific Transcript Profiles Based on Nr-Annotation

To highlight the differences in transcriptional profile of each tissue of *M. galloprovincialis* the similarity between the contiguous sequences from each dataset was determined using an all-against-all BLASTN analysis (Table 2). The digestive gland was most similar to the foot and mantle with ∼10% of digestive gland contigs having a homologue in these two datasets. The foot was the most different tissue, with only 5% of its contigs having homologues with any other tissue. Conversely, 12.5% of gill contigs had homologues in the foot with the digestive gland and mantle containing 9% and 10% homologous contigs respectively. This suggests the gill had the most generalized transcription profile, highlighted by the fact that the mantle had its highest number of homologues with the gill (Table 2).

**Table 2:** Percentage identity between the contiguous fragments of the assembled nucleotide data of each tissue of *M. galloprovincialis*.

		Query			
		Digestive gland	Foot	Gill	Mantle
Reference	Digest gland	100.0	5.7	9.1	6.4
	Foot	10.5	100.0	12.7	7.7
	Gill	5.6	5.5	100.0	8.6
	Mantle	9.1	5.0	10.0	100.0

**Figure 2:**
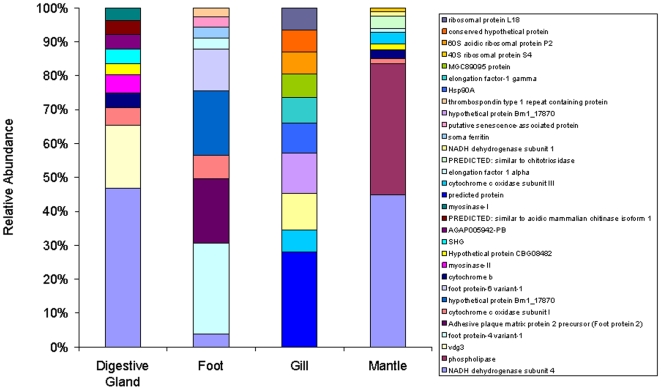
Relative abundance of the top 10 most abundant transcripts within each tissue. Number of reads assembled in to each annotated transcript is normalised based on the number of reads sequenced per sample. Annotation was performed against the NCBI non-redundant database using an e-value cut-off of 1×10^−5^ using BLASTx with assembled reads.

A total of 1023 (12%) of the 8586 contigs could be annotated. Overall, the most abundant annotated transcript was NADH dehydrogenase subunit 4, which comprised 1190 reads (0.7% of total reads) (Fig. 1). The highest abundance of this transcript was in the digestive gland where it constituted 2% of all reads. The second most abundant digestive gland transcript was homologous with vdg3 from *M. edulis* - a developmentally regulated digestive gland-specific marker [14] and importantly, it was only found in the digestive gland (Fig. 2), hence confirming the validity of the high-throughput pyrosequencing approach. In the mantle, NADH dehydrogenase subunit 4 homologues constituted 5 of the top 10 most abundant annotated transcripts. In the foot, 5 of the top 10 most abundant annotated transcripts were homologues of foot proteins identified from *M. californianus and M. galloprovincialis* datasets, specifically adhesive plaque matrix proteins, which constituted 477 reads (1.5% of total). The remaining top 10 foot protein transcripts were annotated as mitochondrial in origin. The top 10 gill transcripts comprised mainly predicted proteins of unknown function or mitochondrial transcripts. A transcript for heat-shock protein, HSP90, which comprised 16 reads was only found in the gill tissues and will have potential use as a gill-specific stress marker.

### Mitochondrial Transcription Profile: Tissue Specific Transcription

**Figure 3:**
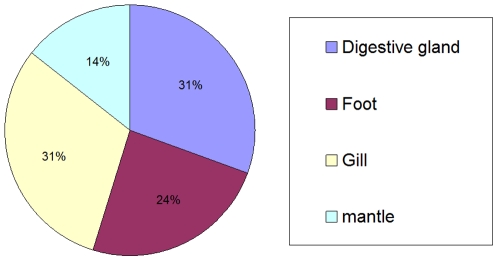
Relative abundance of mitochondrial transcript hits from each tissue. The number of sequences that hit each sample based on 98% nucleotide identity to the mitochondrial genome and is divided by the sequencing effort for each sample.

All raw sequence reads from each tissue sample were aligned against the genome of the relevant mitochondria (Fig. 3). *M. galloprovincialis* shows greatest mitochondrial transcript abundance in the digestive gland and gill, and fewest transcripts in the mantle (Fig. 3). This apparent difference in transcription of genes involved in energy-metabolism between the gill and digestive gland, and the foot and mantle is further evidence of a differential expression in these two tissue groups in accord with expectation.

**Figure 4:**
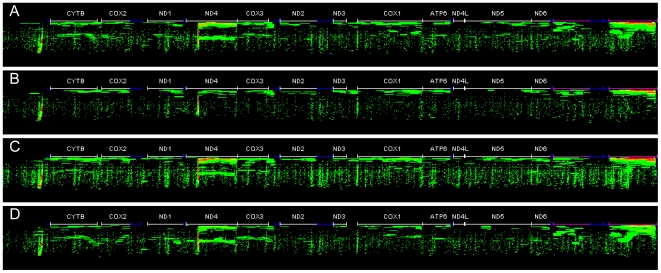
Fragment recruitment heat maps for each sequence dataset based on sequence identity alignment of individual reads to the relevant mitochondrial genome sequence. A–digestive gland; B–foot; C–gill; D–mantle. Purple regions are ribosomal RNA genes and blue regions are potential tRNA sites, white regions are coding sequences; CYTB–cytochrome B, COX2–cytochrome oxidase subunit 2; ND1–NADH dehydrogenase subunit 1; ND4–NADH dehydrogenase subunit 4; COX 3–cytochrome oxidase subunit 3; ND2–NADH dehydrogenase subunit 2; ND3–NADH dehydrogenase subunit 3; COX1–cytochrome oxidase subunit 1; ATP6–ATP synthase subunit 6; ND4L–NADH dehydrogenase subunit 4L; ND5 - NADH dehydrogenase subunit 5; ND6–NADH dehydrogenase subunit 6.

Sequences from each dataset were recruited to the genome of the mitochondria based on sequence identity hits to produce a heat-map of the transcriptional profile of each tissue (Fig. 4). The *M. galloprovincialis* mitochondrial transcriptome shows that the gill and digestive gland have the highest rate of transcript recruitment, with a significantly higher transcription of ribosomal genes. Interestingly, the transcription of NADH dehydrogenase subunit 4 is much higher in the mantle compared to the foot, and yet NADH dehydrogenase subunit 1, 2 and 3 are all higher in the foot than the mantle. This is also true for cytochrome C oxidase subunit 2 and 1, as well as ATP synthase subunit 6. Strikingly, the digestive gland has transcriptional activity in a region not associated with a known protein (upstream of cytochrome b). Indeed all tissues show a low-identity peak in recruitment in this region but the digestive gland has a well defined high-identity transcriptional region (Fig. 4). We hypothesize that the low identity peak in transcriptional activity immediately 5′ of the cytochrome b open reading frame (ORF) could be one of several small RNAs involved in transcriptional regulation in mitochondrial genomes [22].

### The Tissue Specificity of VERL and VCL Transcription

**Figure 5:**
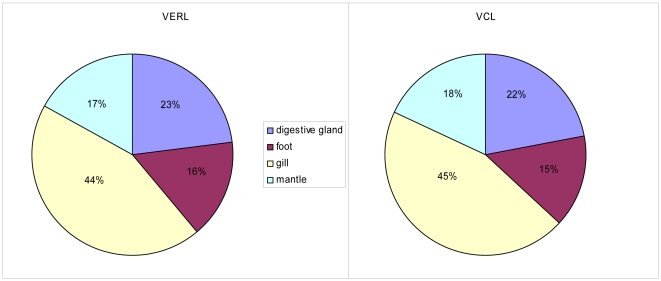
Relative abundance of VCL and VERL transcripts between the different tissues of *M. galloprovincialis*.

**Figure 6:**
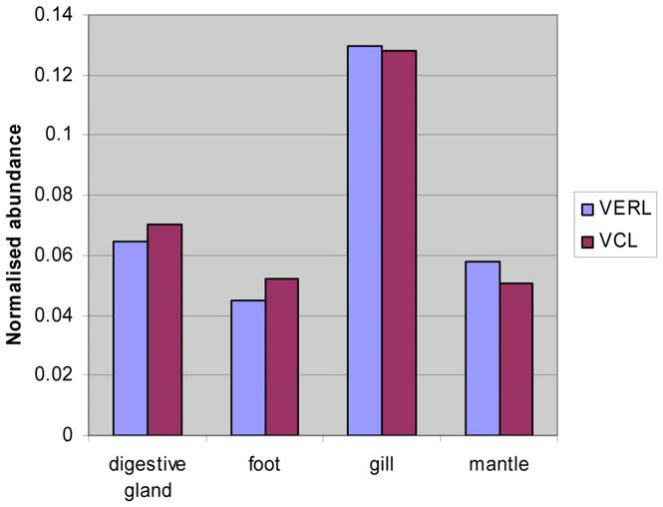
Abundance of hits to the VERL and VCL genes in each sequence database normalised to the sequencing effort of each database.

The abundance of sperm-specific VCL and egg-specific VERL transcript in *M. galloprovincialis* gave a ratio of 0.99 VERL: VCL. This indicates that attempts to provide an equal male to female transcript abundance were successful. Both the VCL and VERL genes are most abundant in the gill (43% and 44% respectively), while expression *in* the foot and mantle show similar levels of transcription to the digestive gland (Fig. 5). Fig. 6 highlights the differential expression of the VERL and VCL genes. In *M. galloprovincialis*, VCL is higher in the digestive gland and the foot, while VERL is higher in the gill and the mantle.

### Overview of Tissue-Specific Differences in Taxonomic and Metabolic Profile

**Figure 7:**
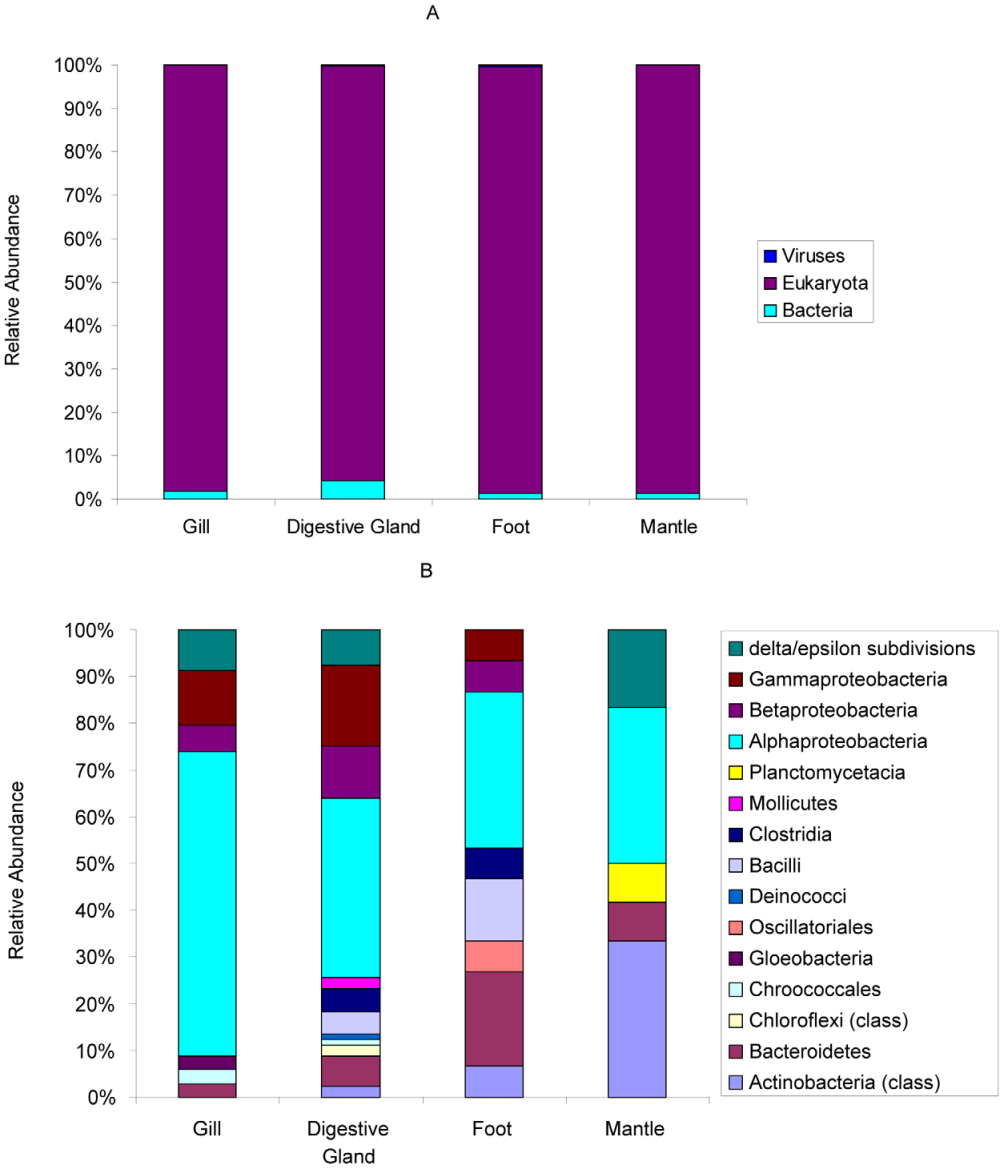
Taxonomic classification of reads in each tissue. Domain-level (A) and bacterial class-level (B) taxonomic distribution of reads for each tissue of *M. galloprovincialis*. Taxonomy annotation is derived from the taxonomy of protein homologue from BLAST alignment with an e-value cut-off of 1×10^−10^ and a minimum alignment of 50 bp.

Taxonomic assignment of individual reads based on homology to known genomes demonstrates that there are tissue specific taxonomic profiles for *M. galloprovincialis* (Fig. 7A). In the digestive gland 4.2% of the transcripts were identified as belonging to bacteria, versus 95.6% as Eukaryotic. As this analysis was performed against known genomes, the Eukaryotic fraction does not contain hits to *Mytilus* as this genome is currently not publically available. There were approximately 3 fold more bacterial transcripts in the digestive gland than in any other tissue, which could be indicative of a greater proportion of commensal bacteria associated with this tissue. Additionally the bacterial species richness (Margalef's d) within the digestive gland was 3.2 compared with an average of 2 for other tissues. This change in diversity as opposed to general community composition is highlighted in Fig. 6B.

**Figure 8:**
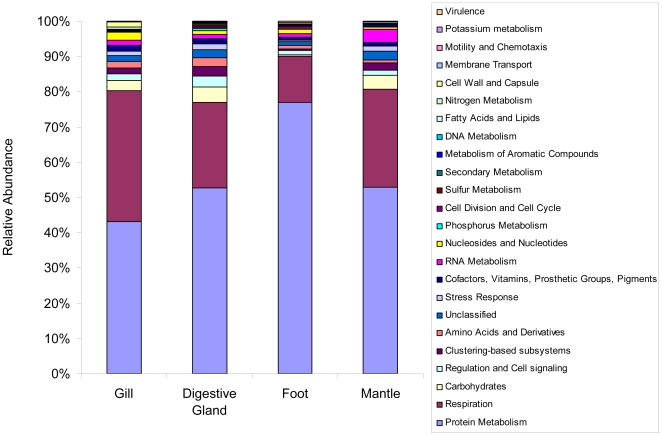
SEED-based subsystem classification of individual raw sequences from each tissue of *M. galloprovincialis*. Annotation is based on BLAST search with an e-value cut-off of 1×10^−10^ and a minimum alignment of 50 bp.

The profile of transcripts related to metabolism in each tissue from *M. galloprovincialis* demonstrates significant similarities and unique tissue-specific differences. The SEED subsystem classification structure was used through MG-RAST annotator to classify sequences into different metabolic groups [23]. There is however, a significant bias towards bacterial sequences in the RAST and MG-RAST database for comparison, hence all hits identified below were explored to make sure that if the RAST homologue was bacterial, that there was the potential for a eukaryotic homologue in the NCBI nr database. This way we were assured that sequence annotation was appropriate for the mussel transcriptome. Based on this classification it can be seen that each tissue is significantly dominated by “protein metabolism” and “respiration”, as would be expected. “Secondary metabolism” is only found in the digestive gland, whereas “motility and chemotaxis” homologues are only found in the foot, and “potassium metabolism” is only identified in the gill tissue (Fig. 8). For all tissues, the most abundant transcripts associated with protein metabolism are the Eukaryotic ribosomal large subunit and small subunit homologues, followed by translation elongation factors. In fact the mitochondrial and Eukaryotic ribosomal transcripts with the elongation factors contribute between 35–40% of the sequences in the digestive gland, gill and mantle, whereas in the foot they contribute ∼70%, highlighting the role of protein synthesis in this tissue. “Respiration” is entirely dominated by cytochrome C oxidase and respiratory complex I transcripts in all tissues. “Carbohydrate metabolism” is the third most abundant pathway, accounting for 3–4% of all transcripts in the gill, digestive gland and mantle but less than 0.5% of transcripts in the foot. Transcripts relating to metabolism of carbohydrates do show significant tissue-specificity. The gill is dominated by methylglyoxal metabolism (involved in the detoxification of the glycolytic byproduct methylglyoxal to d-lactate [24]) and hexitol degradation. While the former is also abundant in the foot and mantle, the latter is virtually absent from any other tissues. Furthermore, the TCA cycle components are abundant in the digestive gland and the mantle, but virtually absent from the gill or foot. In fact the foot only has transcripts involved in methylglyoxal metabolism and D-gluconate and ketogluconates degradation (monosaccharide metabolism). The latter is only found to a small extent in the digestive gland, but overall has its highest transcription in the foot. The foot also has a unique profile with respect to stress response transcripts. Firstly it has the lowest relative transcription of these pathways, and secondly, whereas oxidative stress transcription homologues dominate the other 3 tissues, the foot is dominated by dimethylarginine metabolism, a typical Eukaryotic stress response pathway, which was only found in the foot and mantle. Transcript homologues associated with glutathione redox metabolism were isolated in the digestive gland, and it is known that this system responds in this tissue to environmental pollutants [25].

**Figure 9:**
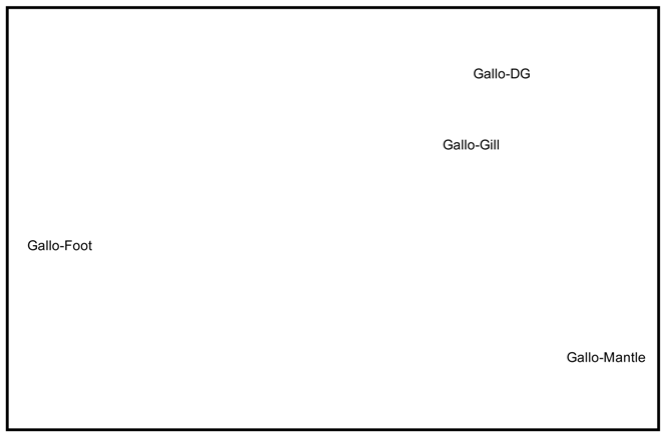
Nonmetric Multi Dimensional Scaling ordination of a Bray-Curtis resemblance matrix among the four different tissues of *M. galloprovincialis* calculated from square-root transformed abundances of 144 unique metabolic subsystems. Each caption represents the composition of the transcriptional profile and the distance between captions is a visual reference to their similarity. 2D stress = 0. DG = digestive gland.

Nonmetric Multi Dimensional Scaling ordination of a Bray-Curtis resemblance matrix of the four different tissues of *M. galloprovincialis* calculated from square-root transformed abundances of 144 unique metabolic subsystems demonstrated that the gill and digestive gland show the closest metabolic similarity. The foot was, as expected, the most dissimilar of the tissues (Fig. 9). The similarity in metabolic profile between the gill and digestive gland could be indicative of their ‘front-line’ position in interactions with the environment.

## Discussion

This work describes the first assessment of the use of pyrosequencing in a mollusk. It has demonstrated the effectiveness of pyrosequencing in rapidly capturing large sections of the Mytilus transcriptome and shown that bar-coding via MID tags can increase productivity, allowing sequences from different tissues to be determined simultaneously but then specifically recovered at the bioinformatics stage. The average read length obtained was low for a typical 454-GS-flx pyrosequencing run, but this are not unprecedented for transcriptomic GS-flx output [26]. The effectiveness of the bar-coding was indicated by the distinctive pattern of transcripts found in each tissue and the limited distribution of some transcripts, e.g. vdg3 only in digestive gland, foot proteins isolated to that tissue, etc. Based on the low number of singletons in most samples a relatively high level of transcriptome coverage is suggested but in a situation where the average number of transcripts per cell in each tissue is unknown, this cannot be confirmed. The genome size of *Mytilus* is estimated to be 1.56×10^11^ bp (http://www.genomesize.com/) so a simplistic guess on the number of genes would be ∼15,000 assuming that the genome does not contain a particularly high proportion of repeat sequences. It is interesting that a recent cloning and sequencing approach has generated ∼9,000 sequences [27]. It is extremely difficult to determine the exact number of unique transcripts generated in pyrosequencing studies, as annotation of fragments is often incomplete leaving a significant number of un-annotated transcripts which would not be included in any assessment. If sequence clustering is used and ORF predicted from these clusters, or if a transcript assembler is used (as described for a portion of this study), an incompletely sequenced transcript may form two or more clusters or assemblies and hence overestimate the total number of transcripts. Hence, no attempt has been made in this study to predict this, although singleton abundance following assembly is a potential proxy for the level of coverage; in theory an absence of singletons would suggest that every transcript has been represented by at least two sequences which form some level of overlap.

The cDNAs used here were not experimentally normalized, so as to allow assessment of transcript abundance and thus it is not surprising to find that transcripts from the mitochondrial genome are prominent. Clear differences in abundance of each mitochondrial gene product between tissues provide a rich and interesting set of observations (Figures 3 and 4). While the biogenesis and transcription of mammalian mitochondria have been extensively studied [28] attention on mitochondria in mussel has centered on Doubly Uniparental Inheritance [5], [29]. In each sample the most abundant of the mitochondrial transcripts were the two ribosomal subunits suggesting that regulation of their transcription is under separate control from the other genes and can proceed from a promoter that is distinct from that used to produce the polycistronic mRNA encoding all of the genes on the heavy strand [28]. This is consistent with the situation in mammals where initiation at the H1 transcription start site, that produces the two ribosomal subunits, is 20 times higher than from the H2 site that produces the polycistronic transcript that is produced from the heavy strand. It is not clear why there should be differential expression of the mitochondrial transcriptome between tissues with higher abundance of transcripts in gill and digestive gland than mantle and foot but this may reflect different physiological roles of the tissues and different energy demands. Thus in gill, energy demand may be high because of beating cilia that are important in filter feeding and because of its role in osmo-regulation. Digestive gland has high energy requirements through its role in intra- and extra-cellular digestion and in detoxification. Differences in levels of mitochondrial transcription between tissues has also been observed in mammals [30]. Within each tissue there is also differential abundance of mitochondrial transcripts which is curious since these are produced initially in polycistronic form. The abundance of each transcript appears to be unrelated to its position on the cistron as illustrated by the finding that in each tissue ND4 was the most abundant mitochondrial mRNA even though it is eleventh in order on the polycistron, while ND6 is present at much lower abundance but is located at position 3 [31]. Differential transcript abundance might be a requirement of the stoichiometry of the electron chain complexes but Complex 1 (NADH ubiquinone oxidoreductase) in mammals contains equimolar quantities of the seven components encoded by the mitochondrial genome [32]. Transcript abundance will be determined not only by rates of transcription but also by differences in nucleolytic processing, by rates of polyadenylation of pre-mRNA and by rates of degradation. These processes will be regulated by intracellular and extracellular factors [30] and are likely to be affected by environmental stressors. Another observation of considerable interest is the identification of mitochondrial transcripts produced from regions not associated with identified genes. These are found in the Control Region (D-loop) and upstream of the Cox1 ORF (Fig. 4). These may be involved in the initiation of DNA replication [28] or control of mitochondrial gene transcription [33].

The tissue distribution of the sex-specific transcripts VERL and VCL are somewhat surprising. It had been expected that these transcripts would be predominantly found in mantle where gametes are produced but very significant quantities were also found in each of the other tissues and indeed are higher in gill than mantle. Previous unpublished work using end-point PCR confirms the occurrence of VERL and VCL in gill and digestive gland (Craft JA and Kennedy J; unpublished). It is unlikely that the occurrence of these transcripts in non-gonadal tissue is a consequence of cross-contamination of tissue resulting from the dissection process since great care was taken at dissection to avoid this and while this may play a role, the similar abundance of transcripts in gill and mantle suggest this is an unlikely explanation. In mussel, reproductive tissue is also found in the visceral mass and mesosoma [34]. Expression of the male-specific protein MAP is also not restricted to the mantle and is also found in foot at levels greater than mantle and at lower levels in digestive gland and gill [35]. Further experimentation including qPCR and in situ hybridization is required to identify those cells in which VERL and VCL are synthesised.

Mytilus are filter feeders and extract plankton and micro-organisms from the water column. The recovery of microbial transcripts predominantly in the digestive gland is consistent with Mytilus physiology. Strikingly, the dominant bacterial transcripts within this tissue come from known pathogens that infect eukaryotes including the tick-borne pathogen *Anaplasma marginale* str. St. Maries and the iron-reducing acidophile *Acidiphilium cryptum* JF-5, both of which were unique to the digestive gland. However, the identification of these bacteria is based on those which have had their genomes sequenced and is therefore significantly biased towards organisms with medical relevance. The recovery of transcript data of this type will provide a valuable tool for marine environmental regulators since it provides clear guides to sources of microbial organisms. In a wider context sequence signals arising from the presence of parasitic and or harmful species such as cyanobacteria, forming shell fish toxins, will be useful for disease assessment of Mytilus. We have previously referred to the potential of such molecular markers in the context of monitoring contamination of organisms [36].

The metabolic profiles show clear tissue-specific differences. Of particular interest was the finding of methylglyoxal metabolism in gill, foot and mantle. This pathway does not appear to have been reported previously in Mytilus species. Methylglyoxal is a by-product of threonine metabolism, lipid peroxidation and glycolytic activity and has cytotoxic and mutagenic effects in mammalian cells [37]. Much current research concerns the consequences to human health of protein glycation by methylglyoxal and the formation of advanced glycation endproducts (AGE) [38]. A further link between the observations made here and in human disease is the relationship between AGE and the iNOS inhibitor dimethylarginine [39] which is also represented in the metabolic profile of foot and mantle.

In conclusion, pyrosequencing in combination with bar-coding has provided extensive genomic information for *M.galloprovincialis*, and amongst other benefits this steps towards the much needed production of an oligonucleotide microarray for the organism and in providing novel observations on expression of different tissues, mitochondria and associated microorganisms

## Methods

### Mussel Collection, Speciation and Sex Determination

Mussels measuring 4–6 cm were collected from intertidal rocks at Port Quin, Cornwall (50°35′16″N, 4°52′19″W) in March 2008. The animals were kept in sea water during transfer back to the laboratory and were dissected within 24 hours. Four tissues (mantle, digestive gland, gill and foot (Fig. 1)) were dissected from 30 individual animals and flash frozen in tubes placed in dry ice and then stored at −80°C until used. DNA was isolated (Nucleospin, ABgene, UK) from individual portions of mantle (25 mg of tissue) prior to species identification using the Me-15 and Me-16 primers and PCR protocol from Inoue et al. [40].

Gender of each mussel was established by a qPCR methodology based upon sequences of the *M. edulis* male-specific Vitelline Coat Lysine (VCL) (Accession#: FM995162) and female-specific Vitelline Envelope Receptor Lysine (VERL) (Accession#: FM995161) genes [41], [42]. The method is described in detail in Text S1.

### Preparation of cDNA Libraries

Mussels were dissected and total RNA isolated from gill, digestive gland, mantle and foot. RNA was extracted from each tissue (50 mg) using the Nucleospin® RNA II Total RNA Isolation Kit (AbGene, UK) following the supplier's protocol. This incorporates a DNase treatment to eliminate genomic DNA. The absence of residual DNA contamination was confirmed by the use of an end-point PCR assay for β-actin (see Supplementary Material) in which a single fragment consistent with the expected size (156 bp) was found. The genomic sequence for β-actin in Mytilus galloprovincialis is not in the database but the actin primers are deemed to sit on either side of an intron and would produce a larger fragment (estimated to be 228 bp) if genomic DNA were present and this has never been seen. The genomic organisation of the Mytilus gene is deduced from that for *Ciona intestinalis* (NW_001955294.1) and *Caenorhabditis briggsae* (CAAC02000521) whose structures are conserved and the high extent of identity of the *M. galloprovincialis* cDNA sequence (AF15749) with that of *Ciona intestinalis* (DQ369967).

Equal quantities of RNA from the four tissues of six male and six female animals (see Text S1 for the method of gender identification) were pooled and the pools used for cDNA synthesis. This was achieved using the SMART™ PCR cDNA Synthesis Kit (Clontech, Paris) using the suppliers protocol [43].

### Pyrosequencing

Individual cDNA libraries were processed as described to allow the addition of MID-labeled primers [44]. The 8 tagged cDNAs were then combined and sequenced on half a picotitre plate using the GS-flx platform (454, Roche, Maryland, USA). MID tags were used to enable subsequent identification of tissue and species specific datasets. Following pyrosequencing, raw sequences were assembled using Newbler V 1.1.03.24.

### Annotation

Assembled contigs were annotated against the National Centre for Biotechnology Information (NCBI) nr protein database using the BLASTx program (version 2.2.1.8) using the default BLOSUM62 matrix. A successful annotation was assigned to a contig if the best hit had an expect-value (*e-*value)≤1×10^−5^. In addition, unassembled sequences were annotated against the SEED-subsystem database through the MG-RAST portal (http://metagenomics.nmpdr.org/; [23]. Sequences were taxonomically classified against SEED based on the taxonomy of protein homologues identified in genome sequences. An *e*-value cut-off of 1×10^−10^ and a minimum alignment length of 50 basepairs were applied. Profiling of metabolism-related transcripts was carried out in the same manner. Sequences are accessible on the MG-RAST website under accession numbers 4442941.3, 4442947.3, 4442948.3, 4442949.3, 4442950.3, 4442952.3, 4442953.3 and 4442954.3.

### Fragment Recruitment

Raw sequence reads were aligned to a fully sequenced mitochondrion from *M. galloprovincialis* using the BLASTN program with match/mismatch scores of 5 and −4 respectively. The top high-scoring segment pair from the best hit was mapped to a fragment recruitment matrix with an x-axis representing the coordinates of nucleotides within the mitochondrion genome and the y-axis representing the identity of the hit. The matrix was then scaled to fit into a 1024 pixel wide image. Due to this scaling, fragments shorter than 17 bp in length do not appear on the recruitment plot as their scaled length is less than 1 pixel wide.

## Supporting Information

Text S1 (0.04 MB DOC)
