# Supplementary material for: Pyrosequencing of Mytilus galloprovincialis cDNAs: Tissue-Specific Expression Patterns
Source: PLoS One. 2010 Jan 25;5(1):e8875. doi: 10.1371/journal.pone.0008875 (PMC2810337; doi:10.1371/journal.pone.0008875)
Supplement: Text S1 — (0.04 MB DOC) [file pone.0008875.s001.doc]

Supplementary Material S1.

Method for Gender Identification of Mussel.

This is based on the male-specific gene Vitelline Coat Lysin (VCL) and the female-specific-gene, Vitteline Envelop Receptor for Lysin (VERL).

RNA was extracted from mantle (50 mg) using the Nucleospin® RNA II Total RNA Isolation Kit (AbGene, UK). cDNA was prepared from this combining total RNA (2 μg), oligo (dT)_12-18_ primer (0.2 μg) and dNTPs (1 mM each) in a final volume of 12 μl. The mix was incubated at 65^o^C for 5 min followed by 5 min on ice. For first-strand synthesis, reaction buffer (1X) and DTT (0.01M) were added. Reactions were incubated at 42^o^C for 2 min followed by the addition of 200 units of SuperScript™ II Reverse Transcriptase (Invitrogen, Glasgow, UK). The reactions were then incubated for a further 50 min at 42^o^C followed by inactivation of the reaction by heating at 70^o^C for 15 min. The prepared cDNA for mussel mantle tissue was then diluted 1:10 using ultra pure water to be used as template for real time q PCR.

qPCR reactions contained cDNA (1μl diluted 1:10), Platinum® SYBR® Green qPCR SuperMix-UDG (Invitrogen, Glasgow, UK), ROX reference dye (1.25µM) with 0.9 μM forward and 0.9 μM reverse primer for either VCL, VERL or actin in separate wells of a 96 well plate (VCL-F 5’ AGAGCTGTTTTGGCCACAGT 3’; VCL-R 5’ TTGCGTTTCACATGGTTGAT 3’ : VERL-F 5’ CCGAAGGAAATGGAACTGAAA 3’ ; VERL-R 5’ CCCTGCAATCGTATGGAATC 3’; Act-f-181me 5’ AGGACTTGTACGCCAACACC 3’; Act-r-182me 5’ CACCGATCCAGACGGAGTAT 3’). Each reaction was made up to a final volume of 20µl. On each run of the qPCR machine (AB 7500) all three genes were screened, in duplicate, in one run for each mussel sample. PCR cycling conditions for each gene were: initial denaturation at 94°C for 15 min followed by 40 cycles of 94°C denaturation for 15 sec, 53°C annealing for 30 sec and 60°C extension for 30 sec. Both VCL and VERL were target genes, with actin being the endogenous control of each experiment. The efficiency of the reactions were: VCL 97.3%; VERL 95.3%; Actin 97.9%. Sex was established by computing for each animal (intra-animal comparison) ΔCt (VERL-VCL) and a negative value indicates female and positive male. ΔCt values of ≤ -3.5 or ≥ +3.5 were considered significant.
